# Rhizosphere-Associated Microbiomes of Rice (*Oryza sativa* L.) Under the Effect of Increased Nitrogen Fertilization

**DOI:** 10.3389/fmicb.2021.730506

**Published:** 2021-09-21

**Authors:** Hangyu Dong, Shuxiu Fan, Haoyuan Sun, Conglin Chen, Aixin Wang, Linlin Jiang, Dianrong Ma

**Affiliations:** ^1^Key Laboratory of Northeast Rice Biology and Breeding, National Rice Regional Technology Innovation Center, Rice Research Institute, Shenyang Agricultural University, Shenyang, China; ^2^College of Agronomy, Shenyang Agricultural University, Shenyang, China

**Keywords:** nitrogen fertilization, rice, rhizosphere, microbiome, chemical properties, enzymes activities

## Abstract

Crops assemble and rely on rhizosphere-associated microbiomes for plant nutrition, which is crucial to their productivity. Historically, excessive nitrogen fertilization did not result in continuously increasing yields but rather caused environmental issues. A comprehensive understanding should be developed regarding the ways in which crops shape rhizosphere-associated microbiomes under conditions of increased nitrogen fertilization. In this study, we applied 16S and 18S ribosomal RNA gene profiling to characterize bacterial and fungal communities in bulk and rhizosphere soil of rice subjected to three levels of nitrogen fertilization for 5 years. Soil biochemical properties were characterized, and carbon-, nitrogen-, and phosphorus-related soil enzyme activities were investigated, by assays. Increasing nitrogen fertilization led to a decreasing trend in the variation of microbial community structures and demonstrated a more definite influence on fungal rather than bacterial community compositions and functions. Changes in the level of nitrogen fertilization significantly affected chemical properties such as soil pH, nutrient content, and microbial biomass levels in both rhizosphere and bulk soil. Soil enzyme activity levels varied substantially across nitrogen fertilization intensities and correlated more with the fungal than with the bacterial community. Our results indicated that increased nitrogen input drives alterations in the structures and functions of microbial communities, properties of soil carbon, nitrogen, and phosphorus, as well as enzyme activities. These results provide novel insights into the associations among increased nitrogen input, changes in biochemical properties, and shifts in microbial communities in the rhizosphere of agriculturally intensive ecosystems.

## Introduction

Soil microorganisms play an essential role in ecosystem function and fertility maintenance by regulating several crucial biogeochemical processes (Fierer, 2017). Plants rely heavily on these microorganisms for nutrient uptake and protection against stresses (Bakker et al., 2018). The rhizosphere is the interface for plant–soil–microbe interactions, which is also impacted by relevant environmental factors, microbiota in the rhizosphere can profoundly affect plant nutrition and growth in an agroecosystem (Philippot et al., 2013). The rhizosphere microbiome extends the functional repertoire of plants and equips host plants with additional gene pools. Therefore, rhizosphere microbiomes are often referred to as second or extended plant genomes (Berendsen et al., 2012; Vandenkoornhuyse et al., 2015). Studies involving *Arabidopsis* (Lundberg et al., 2012), rice (Edwards et al., 2015), and maize (Peiffer et al., 2013) have shown that plant roots selectively recruit specific root microbial communities from the soil. Shifts in the balance between beneficial and harmful microbiota in the rhizosphere might substantially affect crop production in agricultural ecosystems (Chen S. et al., 2019). Therefore, it is of great agronomic interest to understand how crops regulate rhizosphere-associated microbiomes and respond to soil management practices.

Soil enzymes, which combine soil microbial community dynamics with soil nutrient availability, are indicators of microbial function in the soil system (Nannipieri et al., 2018). Soil enzyme activities involved in the transformation of carbon (C), nitrogen (N), and phosphorus (P) play an essential role in soil biochemical processes (Saiya-Cork et al., 2002), indicating the microbiome function in the soil system (Li et al., 2019). The presence of soil enzymes renders soil a complex catalytic system, in which all biochemical transformations depend on enzymatic reactions (Ruggiero et al., 1996). Among the soil hydrolytic enzymes frequently measured, β-glucosidase (BG) is involved in the acquisition of C by hydrolyzing cellulose. Furthermore, N-acetyl-β-D-glucosaminidase (NAG) and leucine aminopeptidase (LAP) play a role in the N-acquisition process by decomposing N-acetyl-β-D-glucosamine or catalyzing the cleavage of amino acids or proteins, respectively. Finally, acid phosphatase (ACP) represents P-acquiring enzymes that hydrolyze phosphate monoesters and organic molecules (Moorhead et al., 2016; Li et al., 2019).

Rice (*Oryza sativa* L.) is a staple food for more than half of the global population, and its perpetual production is crucial for food security (Zhang J. et al., 2019). The application of N fertilizer is one of the most important agricultural practices contributing to increased rice production (Lassaletta et al., 2014). However, there is a great degree of shortsightedness and irrationality in China regarding the excessive use of N fertilizer, which does not improve crop yield and can rather lead to a decline in crop productivity and quality (Ju et al., 2009; Qin et al., 2012). These practices can further result in serious environmental issues, such as groundwater nitrate contamination (Chen et al., 2018), increased greenhouse gas emissions (Liu and Zhang, 2011), and soil acidification (Guo et al., 2010). The overuse of N fertilizer is currently one of the major issues affecting agricultural production in China, particularly in agriculturally intensive areas such as the Northeast Plain (Ju et al., 2009). However, the mechanism by which rhizospheric microbiomes and enzyme activity respond to increased N fertilizer input in rice fields of the Northeast Plain has not yet been investigated. This matter is of great significance for the sustainable development of this intensive agricultural ecosystem.

Establishing the link between the microbial community including bacteria and fungi and enzyme activities in soil – including responses to different levels of N fertilization – has been a long-standing and complex topic in soil ecology, let alone similar investigations in the scenario of flooded rice fields. Problems still exist: (a) what is the response pattern of soil microbial taxonomy, microbial functional composition and soil biochemical properties, including nutrients, microbial biomass and carbon (C), nitrogen (N), and phosphorus (P) related enzymes to excessive N fertilizer application? (b) what are the differences and similarities between bacteria and fungi in response to the increase of N fertilizer application rate? To comprehensively understand these questions, a 5-year field experiment was conducted from 2016 to 2020. Rhizosphere and bulk soil samples were collected from rice plants grown in soil subjected to three levels of N fertilization, 8 weeks after transplantation, once the root microbiota had been well established and stabilized (Edwards et al., 2018; Zhang et al., 2018; Zhang J. et al., 2019). The effect of long-term N fertilization on soil nutrient availability and transformation was assessed by determining properties of C, N, and P, as well as enzyme activities in soil. Bacterial and fungal communities in rhizosphere and bulk soil were determined using PacBio and Illumina Novaseq sequencing platform. The results of this study provide in-depth information on the bacterial and fungal communities, soil properties, and C-, N-, and P-associated enzyme activities in soil in response to increased N fertilization. Efficient, continuous crop production can only occur with sound agricultural practices, which depend on an adequate understanding of the impact of those practices on the resources available for agriculture. We believe that our study makes a significant contribution to the literature because it aids in expanding this required understanding while creating an opportunity for further related research.

## Materials and Methods

### Field Experiment and Sample Collection

A 5-year N fertilization field experiment was initiated in 2016 at the research farm of Shenyang Agricultural University in Shenyang City, Liaoning Province, China (41°49′ N, 123°34′ E). Average annual temperature and precipitation in the area are 8.4°C and 715.5 mm, respectively. We tested typical paddy soil, in which rice had been planted for more than 30 years. The experimental land was demarcated into plots, 8.6 m × 9 m in size. A Japonica rice variety – Liaoxing 1 (CNA20040621.3) – was provided by the Rice Research Institute of Shenyang Agricultural University and used in experimental planting; this cultivar, with its stable yield, moderate growth cycle, and strong resistance, is widely planted in Northeast China. The experiment evaluated three levels of N fertilization – 0, 150, and 300 kg/hm^−2^ – per rice-growing season. According to the rice production management practice, the recommended nitrogen application rate of Liaoxing 1 was 150 kg hm^–2^ during the growing season, and the N application treatments selected in this study were 100% and 200% of the recommended N application rate, respectively. Each treatment was replicated three times; hence, nine experimental plots were completely randomized and surveyed during the experimental period. Urea (N: 46%), as the only type of nitrogen fertilizer was applied in three sessions, i.e., 50% before transplanting, 30% at tillering stage, and 20% at panicle initiation stage. The application rates of P (phosphorus, calcium superphosphate, 375 kg/hm^–2^) and K (potassium, potassium chloride, 112.5 kg/hm^–2^) fertilizers were the same in all experimental plots, and 100% P and K fertilizers were applied before the transplanting. Rice seeds were disinfected – soaked in 0.01% potassium permanganate solution for 24 h – and then seeded were sown on the substrate soil of rice seedlings in the greenhouse. The substrate can meet the requirements of nutrients for seeding growth. The environment of the greenhouse was very suitable for the growth of rice seedlings with the temperature maintained at 25–27°C. It was ventilated twice a day to keep sufficient oxygen in the greenhouse. After 1 month of growth in the greenhouse, the rice seedlings were transplanted into the experimental field. This experimental design was consistently applied from 2016 to 2020. To prevent fertilizer leakage, different N fertilizer treatment plots were separated by PVC partitions and artificial ridges. The distance between plots was 30 cm, rice on the boundary of plots were designed for protection and not sampled. In order to promote rooting and prevent ineffective tillers, a week of water control management was carried out at tillering stage (early July). Irrigation in paddy field was carried out after water control, and the remaining growth period of rice was completed under flooded condition. During this period, field management shall be carried out according to local field management practice. Rhizosphere and bulk soil samples were collected at the full heading stage, 8 weeks after transplantation of the rice seedlings (2020). At this stage, the rhizosphere associated microbiota had been well established and stabilized. Another reason why we chose to take samples in this period is that for the growth period of Liaoxing 1, the plant was in the heading and filling period at this time. This period is important for rice growth and development, which directly affects the grain yield at the harvest stage. Therefore, it is of great significance to explore the associated microorganisms and soil biochemical properties in this period. Three replicate samples of rhizosphere and bulk soil were collected from plants (randomly select 12–15 rice plants in each plot) subjected to each of the three N fertilization levels. Each complete rice plant was removed with its immediate block of soil included. The soil loosely attached to the roots was collected as bulk soil samples, whereas remaining soil on the root surfaces was brushed off gently with a sterile brush and gathered as rhizosphere soil samples (Chen S. et al., 2019).

### DNA Extraction and Amplicon Sequencing

We used the NucleoSpin 96 Soil kit (740787.2, Macherey-Nagel, Düren, Germany), as per the manufacturer’s protocol, to extract total genomic DNA from rhizosphere and bulk soil samples. Furthermore, we tested the integrity, concentration, and purity of DNA using electrophoresis on a 1% agarose gel. The 16S and 18S rRNA genes were amplified with primers 27F:1492R (AGRGTTTGATYNTGGCTCAG/TASGGHTACCTTGTTASG ACTT) and ITS1F/ITS2 (5′-CTTGGTCATTTAGAGGAA GTAA-3′/5′-GCTGCGTTCTTCATCGATGC-3′), respectively. PCR amplification was performed in a 20 μl mixture containing 1 μl VnF (10 μM) and 1 μl VnR (10 μM), 1 μl DNA template (approximately 5–50 ng of DNA), 0.4 μl KOD FX Neo (TOYOBO), 10 μl KOD FX Neo Buf, and 4 μl 2 mM dNTP and then supplemented with 20 μl ddH_2_O. The PCR conditions were as follows: 95°C for 5 min; 25/30 cycles of 30 s at 95°C, 30 s at 50°C, and 1 min at 72°C, with a final extension at 72°C for 7 min. The products were examined using agarose gel electrophoresis and purified using AMPure XP beads (Beckman Coulter, Inc., Brea, CA, United States), as per the manufacturer’s protocol. The purified PCR products were quantified and homogenized to form a sequencing library, which was then subjected to library quality inspection; after passing the quality inspection, the library was sequenced using the PacBio Sequel System (Biomark Biotechnology Co., Ltd., Beijing, China).

Sequencing data analysis was divided into three steps. First, Lima v1.7.0 software^1^ was used to identify circular consensus sequences (CCSs) through barcodes, and obtain barcode-CCS sequence data. Second, the barcode-CCSs were filtered to obtain valid sequences, and third, UCHIME v4.2 software^2^ was used to identify and remove the chimera sequences in to obtain the optimization-CCS sequence. Sequence data were clustered into operational taxonomic units (OTUs) at a 97% similarity level using the USEARCH^3^ method. Species annotation and abundance analyses were performed using bacterial and fungal 16S and 18S rRNA datasets from the SILVA database to reveal the species compositions of samples. The raw FASTQ files obtained in the study for the sequencing libraries have been deposited into NCBI Sequence Read Archive (SRA) under BioProject accession numbers PRJNA725973 (bacterial) and PRJNA725981 (fungal).

### Soil Chemical Properties

The soil samples were mixed with distilled water at a ratio of 1:2.5 to remove CO_2_. After 30 min, the pH was measured using a pH meter (Mettler Toledo, FP20). We further used the potassium dichromate external heating method to measure total organic carbon (TOC), whereas soil total nitrogen (TN) was measured using the semi-micro Kelvin method (Lu, 2000). Available nitrogen (AN) was measured using the alkaline hydrolysis diffusion method, and available phosphorus (AP) was measured using the molybdenum-antimony anti-colorimetric method (Lu, 2000). Microbial biomass C and N were determined using the chloroform fumigation-K_2_SO_4_ extraction method, and microbial biomass P was determined by the chloroform fumigation-NaHCO_3_ extraction method (Bao, 2000). In short, the soil sample was fumigated with chloroform steam in a vacuum desiccator for 24 h, and residual chloroform was removed by repeated vacuuming. After adding 0.5 M K_2_SO_4_ or NaHCO_3_, respectively, the mixture was shaken for 30 min and filtered immediately. The filtered extract was used to determine microbial biomass C, N, and P. A TOC analyzer was used to determine microbial biomass C, whereas a flow injection analyzer was used to determine microbial biomass N. Mo-Sb colorimetry was used to determine microbial biomass P.

### Soil Enzyme Activities

Enzyme activity levels in soil samples was investigated using a double-antibody sandwich enzyme-linked immunosorbent assay kit (MEIMIAN, Jiangsu Meimian Industrial Co., Ltd.), as per the manufacturer’s instructions. The weight of soil used to determine the activity of enzyme was 1 g. Then the soil was evenly divided into two parts, one part was used to measure the enzyme activity and the other part was for the determination of water content (drying method). Considering LAP as an example, the microplate was coated with purified LAP antibody (Catalog Number: HZ74634) to prepare a solid-phase antibody; LAP was then added to the microwells of the coated monoclonal antibody, before being combined with horseradish peroxidase (HRP)-labeled LAP antibody, to form an antibody–antigen–enzyme-labeled antibody complex. After thorough washing with deionized water, tetramethylbenzidine (TMB; MEIMIAN, Jiangsu Meimian Industrial Co., Ltd.) was added for color development. TMB turned blue in response to the catalytic activity of HRP (Catalog number, MEIMIAN, Jiangsu Meimian Industrial Co., Ltd.) and then yellow after the addition of acid (sodium acetate, citric acid). Color change was measured spectrophotometrically at a wavelength of 450 nm. The color intensity was positively correlated with the activity of LAP in the sample, and then the activity level of LAP in the sample was determined by comparing the OD (optical density) value of the samples to the stand curve. Activity levels of NAG, BG, and ACP (Catalog numbers for antibodies, NAG: HZ69087, BG: HZ70938, and ACP: HZ87604, respectively) were measured in a similar manner.

### Statistical Analyses

Statistical analyses were performed using SPSS Statistics, Version 23 (IBM Corp., Armonk, NY, United States). Analysis of variance (ANOVA) and least significant difference analyses were used to test the significance of the effect of increased N fertilization on soil chemical properties and enzyme activity. Bioinformatics analyses were performed using BMKCloud^4^, principle coordinate analysis (PCoA) and analysis of similarities were performed to determine the differences in microbial community structure, and alpha diversity analysis was performed to study microbial community richness and diversity. We annotated bacterial functions using the FAPROTAX database (Louca et al., 2016), based on currently available literature on cultivated strains. FUNGuild (Nguyen et al., 2016) was used to predict the ecological functions of fungal communities, as a tool for linking fungal gene sequence information – obtained by high-throughput or cloning libraries – with fungal functions. Finally, redundancy analysis (RDA) was performed to explore the relationships between microbial communities and soil enzyme activities.

## Results

### Grain Yield of Rice

**FIGURE 1:**
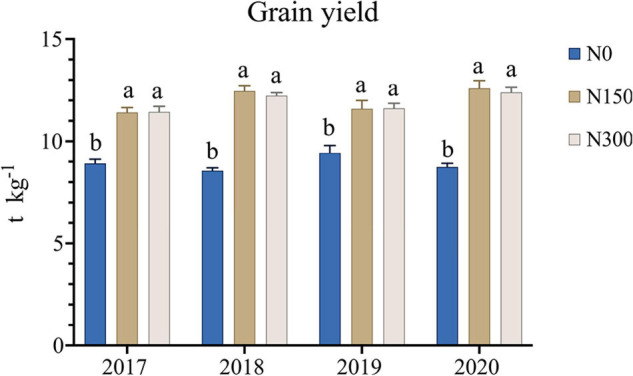
The grain yield of Liaoxing 1 under the three N fertilizer application levels from 2017 to 2020. Different lowercase letters for one data indicate a significant differences at *p* < 0.05.

We recorded the grain yield data of Liaoxing 1 from 2017 to 2020, and the results are shown in Figure 1. The 4-year yield data showed that the rice yield under nitrogen application treatment (N150 and N300) was significantly (*p* < 0.05) higher than that without N application treatment (N0), but the rice yield under nitrogen application treatment N150 and N300 had no significant difference (*p* > 0.05). In 2018 and 2020, the rice yield showed a downward trend with the doubling of N fertilizer application.

### Structure of Bacterial and Fungal Communities

**FIGURE 2:**
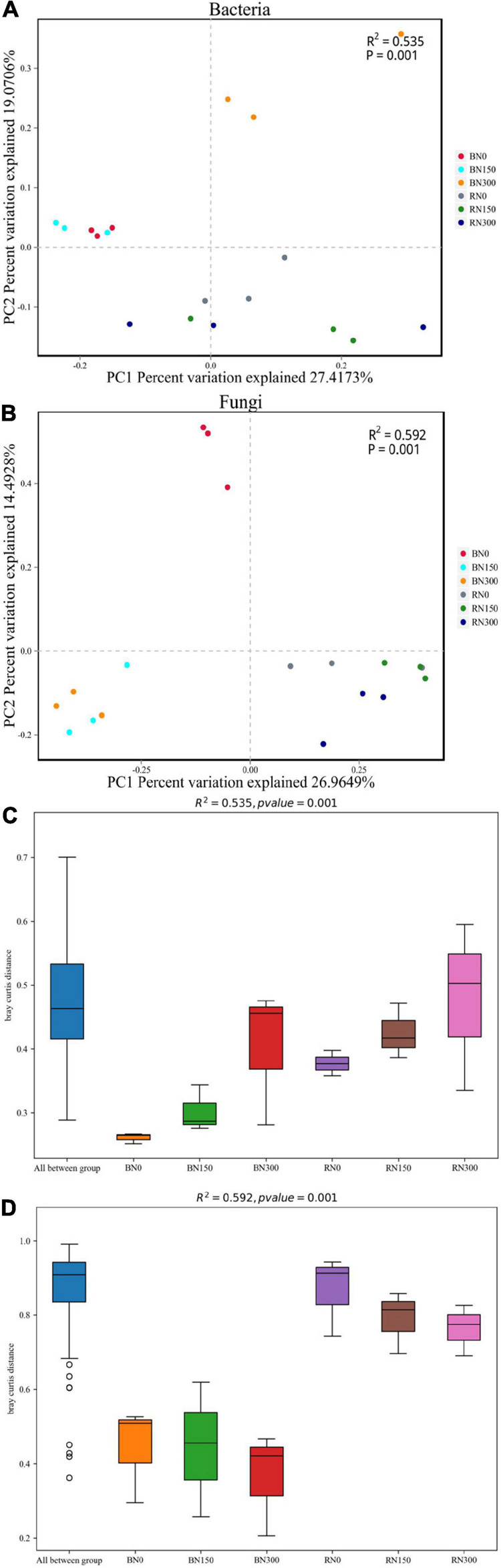
Principle coordinate analysis (PCoA) of the bacterial **(A)** and fungal **(B)** communities in rhizosphere and bulk soil samples. Dissimilarity distances showing the differences in bacterial **(C)** and fungal **(D)** communities, in rhizosphere and bulk soil samples. PCoA and dissimilarity distances were based on the Bray-Curtis distance algorithm at the OTU level. BN0 and RN0 represent the bulk soil and the rhizosphere soil of rice under N0 treatment; BN150 and RN150 represent the bulk soil and the rhizosphere soil of rice under N150 treatment; BN300 and RN300 represent the bulk soil and the rhizosphere soil of rice under N300 treatment.

Principle coordinate analysis based on the Bray-Curtis distance algorithm was performed at the OTU level (Figures 2A,B). The OTUs of bacteria and fungi were separated per rhizosphere and bulk soil samples for each of the three N treatment levels. In bulk soil samples, bacterial OTUs from soil subjected to N0 and N150 treatments were more similar to each another and distinct from the OTUs from soil treated with N300. Simultaneously, the fungal OTUs from N150- and N300-treated soil were more similar to one another but distinct from the OTUs from N0-treated soil. The OTU distances of bacteria and fungi in rhizosphere samples from soil subjected to all three N treatments were all relatively uniform. Dissimilarity distances among the three N fertilization levels were calculated across the bulk and rhizosphere soil samples (Figures 2C,D). At similar N application levels, the differences in bacterial and fungal community structures from bulk to rhizosphere soil showed an increasing trend. Furthermore, with increased N application, differences in bacterial community structures showed an increasing trend, whereas differences in the fungal community showed a decreasing trend.

### Diversity and Richness of Bacterial and Fungal Communities

We evaluated the alpha diversity of each sample using the Shannon and Chao1 indices to measure species diversity and richness, respectively (Table 1). We found no significant (*p* > 0.05) difference in bacterial species diversity between rhizosphere and bulk soil samples, whereas fungal species diversity in rhizosphere soil samples was significantly (*p* < 0.05) higher than that in bulk soil samples. Notably, the species richness of both bacteria and fungi in rhizosphere soil samples was significantly (*p* < 0.05) higher than that in bulk soil samples.

**TABLE 1:** The diversity and richness of bacterial and fungal communities in rhizosphere and bulk soil samples subjected to three levels of nitrogen (N) fertilization, characterized by Shannon and Chao1 indices according to alpha diversity analysis.

	**N treatment level**	**Shannon index of bacteria**	**Shannon index of fungi**	**Chao1 index of bacteria**	**Chao1 index of fungi**
	N0	9.50a	5.08c	1907.85a	542.98a
**Rhizosphere samples**	N150	9.21b	6.03a	1796.28b	546.33a
	N300	9.09c	5.51b	1705.77d	486.45b
	N0	9.34b	3.90d	1749.70c	461.47b
**Bulk soil samples**	N150	9.31b	3.02f	1669.39e	389.88c
	N300	8.89c	3.47e	1642.05f	317.83d

*Different lowercase letters following one data point indicate significant differences at *p* < 0.05.*

With increased N application, bacterial species diversity and richness in both the rhizosphere and bulk soil samples showed a significant (*p* < 0.05) decrease, particularly in soil subjected to the N300 treatment, it was significantly lower than that in N0- and N150-treated soil. With increasing N application, fungal species diversity in rhizosphere soil samples increased at first and then decreased, whereas an opposite pattern was observed in the bulk soil samples. Furthermore, the species richness of fungal communities in both rhizosphere and bulk soil samples showed a significantly (*p* < 0.05) decreasing trend with increasing N application.

### Species Composition of Bacterial and Fungal Communities

**FIGURE 3:**
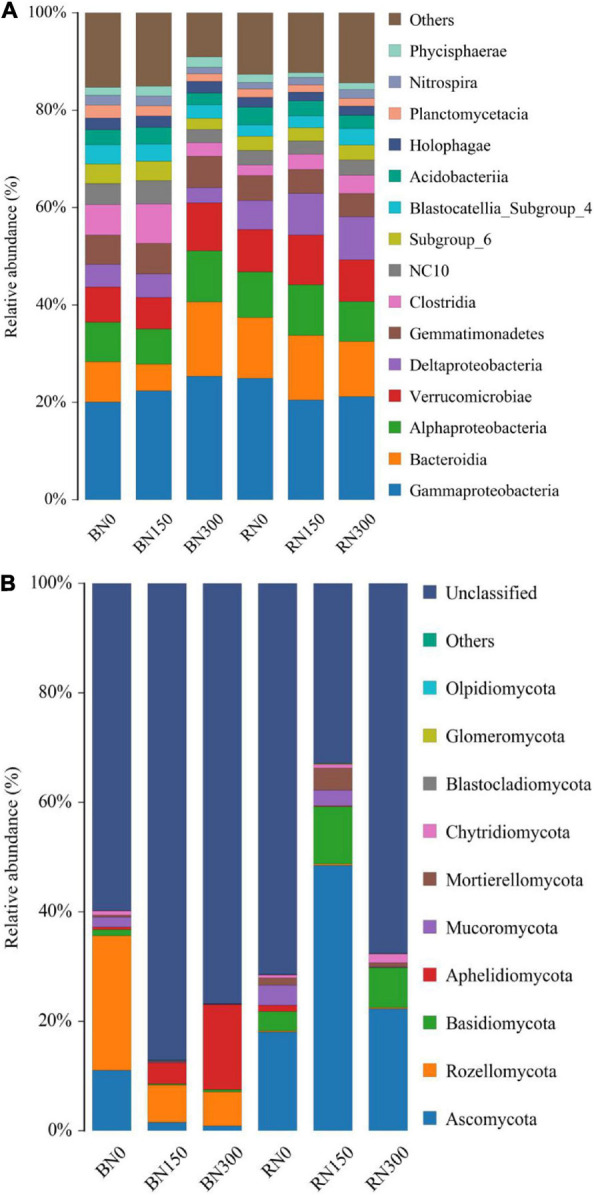
Bacterial **(A)** and fungal **(B)** community composition of rhizosphere and bulk soil samples subjected to the three levels of N fertilization.

This study also evaluated the bacterial and fungal species composition from phylum to family level in rhizosphere and bulk soil samples subjected to the three N fertilization treatment alternatives. Considering bacteria, increasing the levels of N application resulted in an increasing trend in the relative abundance of Gammaproteobacteria in bulk soil samples, whereas the relative abundance of Bacteroidia decreased initially and then increased. The relative abundances of Deltaproteobacteria, Clostridia, NC10, and Nitrospira were the lowest in soil subjected to N300 treatment. In rhizosphere samples, the relative abundance of Nitrospira showed an increasing trend, whereas that of Gammaproteobacteria showed a decreasing trend, in line with the increased N application. The relative abundances of bacteria (at class level) in rhizosphere and bulk soil samples subjected to increasing levels of N fertilization application are shown in Figure 3A.

Figure 3B compares the composition of fungal communities (at phylum level) in rhizosphere and bulk soil samples in response to increasing N fertilization. In bulk soil samples, the relative abundance of Ascomycota decreased significantly with an increase in N fertilization, and the relative abundance of Rozellomycota decreased by 72.2% in N150 samples and 74.7% in N300 samples, compared to levels in soil subjected to N0 treatment. Furthermore, the relative abundance of Aphelidomycota increased dramatically by 875.0% in N150 soil and 3200.0% in N300 soil, respectively, compared to levels in N0 soil. In rhizosphere samples, the relative abundance of Ascomycota, Basidiomycota, and Mortierellomycota increased initially and then decreased, whereas the relative abundance of Mucoromycota decreased outright, with increasing N fertilization. We further found that fungal populations – such as Rozellomycota and Aphelidomycota – that were selectively enriched in the rhizosphere were detected in higher proportions in bulk soil samples than in rhizosphere soil samples, where they were hardly annotated. Conversely, Ascomycota and Basidiomycota accounted for a higher proportion of fungal populations detected in rhizosphere samples. A comparison of the bacterial and fungal community compositions in rhizosphere and bulk soil samples revealed that the microbial rhizosphere effect of rice plants was mainly reflected in the selective enrichment of certain fungal communities.

### Functions of Bacterial and Fungal Communities

**FIGURE 4:**
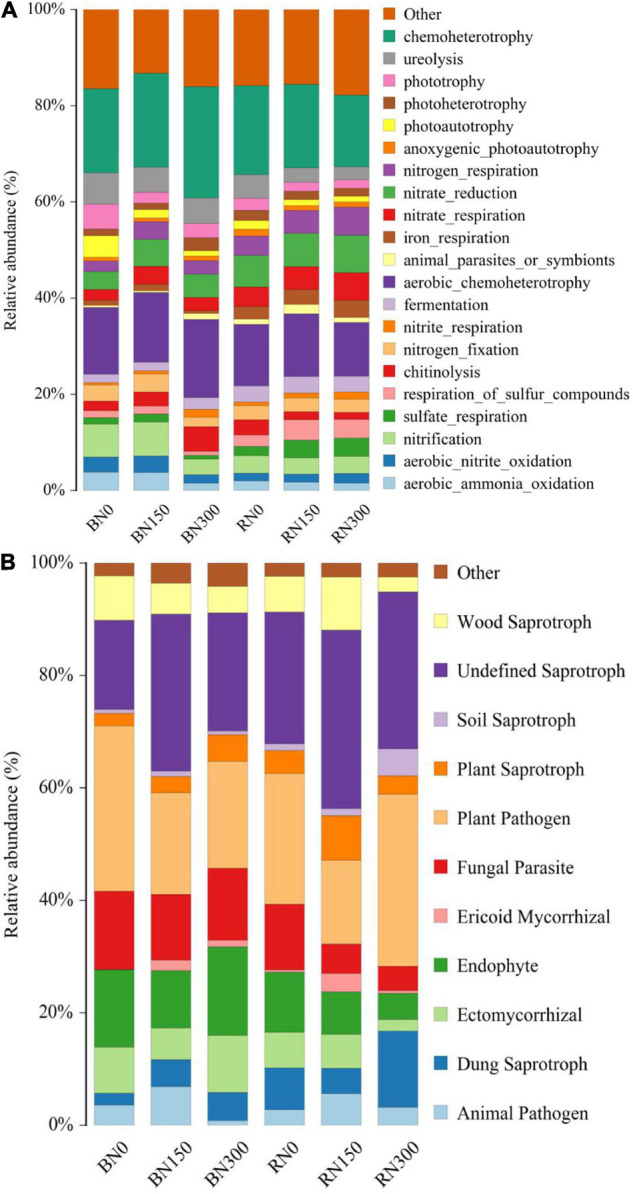
The functions of bacterial communities and ecological functions of fungal communities, were explored using FAPROTAX **(A)** and FUNGuild **(B)**, respectively.

Increased N fertilizer input affected the functions of bacterial (Figure 4A) and fungal (Figure 4B) communities in both rhizosphere and bulk soil samples. In soil subjected to N300 treatment, bacterial aerobic nitrate oxidation, aerobic ammonia oxidation, nitrification, and N fixation were reduced, whereas chitinolysis was enhanced. Increased N fertilization had a more negligible effect on the functions of bacteria in the rhizosphere compared to that in bulk soil, mainly reflected by enhanced sulfate and sulfide metabolism, as well as diminished degradation of chitin and urea, in rhizosphere samples. Considering the fungal players in our study, increased N fertilization resulted in an initial increase, followed by a decrease, in the relative abundance of fungi with animal pathogens and ericoid mycorrhizal functions in both rhizosphere and bulk soil samples. With an increase in N fertilization from N150 to N300, the relative abundance of fungi with ectomycorrhizal functions showed an increasing trend in bulk soil samples and a decreasing trend in rhizosphere samples. Notably, the relative abundance of plant pathogens in the rhizosphere was highest in soil subjected to the N300 treatment, indicating that the adverse effects of high N fertilization on the plant might be associated with an accumulation of plant pathogens.

### Response of Soil Properties to Increased N Fertilization

Characteristics of soil pH, TOC, TN, AN, and AP are shown in Table 2. Our findings were as follows: increased N application significantly reduced the pH of both the rhizosphere and bulk soil, with rhizosphere pH being significantly lower than that of bulk soil, especially in soil subjected to N150 and N300 treatments. Furthermore, in soil subjected to N150 treatment, the TOC content of rhizosphere soil samples was significantly lower than that of bulk soil samples, whereas the opposite trend was observed in soil subjected to N300 treatment. With an increase in N fertilization, the TOC content of the rhizosphere soil samples increased overall, whereas the TOC content of bulk soil samples increased initially and then decreased. A significantly higher TN level was observed in rhizosphere soil samples subjected to N0 treatment than in N150- and N300-treated soil. Conversely, the TN content in bulk soil samples was significantly lower in N0-treated soil than in soil subjected to the two higher levels of N treatment. Furthermore, the TN content in N150- and N300-treated bulk soil samples was significantly higher than that in equivalent rhizosphere soil samples. The AN content in N0- and N150-treated rhizosphere soil samples was higher than that in comparative bulk soil samples but significantly lower in soil subjected to N300 treatment. The AN content of both rhizosphere and bulk soil samples increased after N150 treatment and then decreased after N300 treatment. Finally, the AP content of N0-treated rhizosphere soil samples was significantly lower than that of similar bulk soil samples, whereas no significant difference was observed in the AP content between the rhizosphere and bulk soil samples subjected to N150 and N300 treatments.

**TABLE 2:** Soil pH and carbon (C)-, nitrogen (N)-, and phosphorus (P)-nutrient contents of rhizosphere and bulk soil samples subjected to the three nitrogen (N) fertilization levels.

	**N treatment level**	**pH**	**TOC (g kg^–1^)**	**TN (g kg^–1^)**	**AN (mg kg^–1^)**	**AP (mg kg^–1^)**
	N0	6.19 ± 0.06ab	12.02 ± 0.27e	1.54 ± 0.11c	15.29 ± 0.34c	22.97 ± 1.94c
**Rhizosphere samples**	N150	5.36 ± 0.19d	13.24 ± 0.27c	1.24 ± 0.18d	19.10 ± 0.25a	26.74 ± 1.09ab
	N300	5.07 ± 0.13e	13.72 ± 0.18b	1.35 ± 0.06d	12.97 ± 0.12d	24.66 ± 0.86bc
	N0	6.34 ± 0.14a	12.26 ± 0.29de	1.32 ± 0.07d	13.30 ± 0.44d	29.21 ± 1.50a
**Bulk soil samples**	N150	6.02 ± 0.03b	14.97 ± 0.07a	1.70 ± 0.19b	17.67 ± 0.22b	27.73 ± 2.62ab
	N300	5.61 ± 0.03c	12.54 ± 0.18d	2.13 ± 0.33a	15.88 ± 0.31c	27.97 ± 2.92ab

*TOC, total organic carbon; TN, total nitrogen; AN, available nitrogen; AP, available phosphorus. Different lowercase letters after one data point indicate significant differences at *p* < 0.05.*

Next, we measured microbial biomass carbon (MBC), nitrogen (MBN), and phosphorus (MBP), the results of which are presented in Table 3. MBC and MBN in the rhizosphere and bulk soils were generally not significantly affected by increased N fertilization, based on the outcomes of ANOVA. However, MBC in soil subjected to N300 treatment was significantly lower than that in N0- and N150-treated bulk soil samples. Nevertheless, we identified some specific trends, including an initial increase in rhizospheric MBC and MBN content followed by a decrease in response to increased N fertilization. Furthermore, as N fertilization increased in bulk soil samples, MBC content increased at first before decreasing, whereas MBN first decreased and then increased. In terms of MBP, opposing trends were observed in the responses to increased N fertilization between rhizosphere and bulk soil samples; a decreasing shift was observed in rhizosphere samples, with an increasing shift in bulk soil samples.

**TABLE 3:** Soil microbial biomass content of rhizosphere and bulk soil samples subjected to the three nitrogen (N) fertilization levels.

	**N treatment level**	**MBC (mg kg^–1^)**	**MBN (mg kg^–1^)**	**MBP (mg kg^–1^)**
	N0	98.55 ± 14.12a	38.92 ± 4.81abc	23.62 ± 3.94a
**Rhizosphere samples**	N150	90.62 ± 17.90ab	45.60 ± 7.56a	21.55 ± 6.46a
	N300	81.34 ± 6.33ab	40.87 ± 1.52ab	13.04 ± 1.15b
	N0	80.14 ± 11.69ab	34.87 ± 6.76bc	13.59 ± 2.19b
**Bulk soil samples**	N150	99.87 ± 15.29a	29.34 ± 6.97c	17.53 ± 1.42ab
	N300	62.42 ± 7.76c	31.80 ± 2.95bc	23.16 ± 1.53a

*MBC, microbial biomass carbon; MBN, microbial biomass nitrogen; MBP, microbial biomass phosphorus. Different lowercase letters after one data point indicate significant differences at *p* < 0.05.*

### Response of Soil Enzyme Activities to Increasing Levels of N Fertilization

The activities of four key enzymes related to C, N, and P are shown in Table 4. We found that the activities of N-acquiring enzymes (NAG, LAP) in rice rhizosphere soil were higher than those of C- (BG) and P-acquiring (ACP) enzymes. By comparing soils subjected to the three levels of N treatment, BG, NAG, and LAP activities in rhizosphere soil samples were significantly higher than those in bulk soil samples. In contrast, ACP activity in rhizosphere samples was significantly lower than that in bulk soil samples. Furthermore, BG and LAP activities in both rhizosphere and bulk soil samples subjected to N300 treatment were significantly lower than those in N0- and N150-treated soil. Conversely, NAG activity was higher in N300-treated soil than in N0- and N150-treated soil. ACP activity was significantly higher in rhizosphere soil samples subjected to N300 treatment compared with to that in N0- and N150-treated soil, whereas no significant difference was observed in ACP activity among bulk soil samples subjected to the three N treatment levels.

**TABLE 4:** Activity levels of carbon (C)-, nitrogen (N)-, and phosphorus (P)-acquiring enzymes in rhizosphere and bulk soil samples subjected to the three nitrogen (N) fertilization levels.

	**N treatment levels**	**BG (IU g^–1^)**	**NAG (IU g^–1^)**	**LAP (IU g^–1^)**	**ACP (IU g^–1^)**
	N0	8.84 ± 0.21a	54.54 ± 0.35c	33.69 ± 0.38a	6.90 ± 0.28cd
**Rhizosphere samples**	N150	8.91 ± 0.12a	58.75 ± 1.61b	31.91 ± 0.97b	6.73 ± 0.21d
	N300	7.88 ± 0.21b	61.84 ± 2.63a	28.23 ± 0.48d	8.80 ± 0.23a
	N0	7.78 ± 0.14b	44.83 ± 1.14e	29.6 ± 0.93c	7.47 ± 0.11b
**Bulk soil samples**	N150	7.11 ± 0.19c	39.45 ± 0.76f	28.17 ± 0.58d	7.39 ± 0.13b
	N300	6.64 ± 0.11d	50.03 ± 1.21d	24.63 ± 0.79e	7.21 ± 0.09bc

*BG, 1,4-β-glucosidase; NAG, 1,4-β-N-acetylglucosaminidase; LAP, leucine aminopeptidase; ACP, acid phosphatase. Different lowercase letters after one data point indicate significant differences at *p* < 0.05.*

**FIGURE 5:**
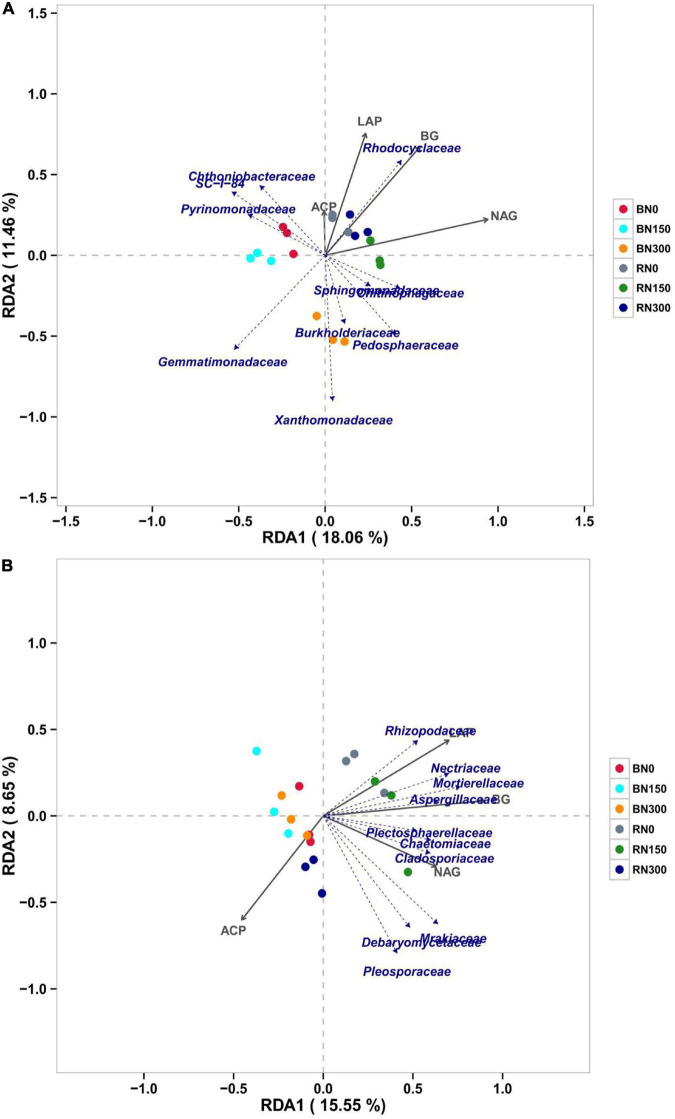
Correlation between bacterial **(A)** and fungal **(B)** communities and soil enzyme activities explored using RDA (redundancy analysis).

We conducted RDA on microbial communities and soil enzyme activities (Figure 5) at the family level to explore the correlation between microbial communities and nutrient-acquiring enzymes in soil. Considering the bacterial communities (Figure 5A), Rhodocyclaceae was positively correlated with LAP, BG, NAG, and ACP activities, indicating that the bacteria could increase enzyme activities related to rhizospheric C, N, and P. Furthermore, we found that Gemmatimonadaceae and Xanthomonadaceae were negatively correlated with the activities of the four nutrient-acquiring enzymes and had a higher correlation with the composition of the microbial community in soil treated with N300, indicating that it is easier for these two bacteria to colonize bulk soil with high N fertilization. With regard to fungi (Figure 5B), we found that the majority were negatively correlated with ACP activity, indicating that the presence of fungi could reduce ACP activity. Positive correlations were observed between Rhizopodaceae and LAP activity, Nectriaceae, Mortierellaceae, or Aspergillaceae and BG activity, and Plectosphaerellaceae, Chaetomiaceae, or Cladosporiaceae and NAG activity, indicating that these fungal communities might positively affect NAG, LAP, and BG activities, respectively. Moreover, we found that bacterial and fungal genera related to soil enzyme activities were more significantly correlated in rhizosphere than in bulk soil samples.

## Discussion

The excessive use of N fertilization is currently one of the main problems affecting agricultural production in China (Ha et al., 2015), particularly in agriculturally intensive areas such as the Northeast Plain. In this study, we found that doubling the amount of N fertilizer would not result in a significant increase in grain yield of rice (Figure 1), which was consistent with the previous studies (Ju et al., 2009; Niu et al., 2017), indicating that excessive use of N fertilizer exceeding a certain threshold will not increase crop yields, but may lead to N losses and serious environmental problems. Therefore, improving the N-use efficiency of crops and reducing N fertilization are the main challenges in achieving sustainable agricultural development (Chen J. et al., 2019). The crucial role of bacteria and fungi in nutrient turnover in the rhizospheric zone (Chen S. et al., 2019) necessitates comprehensive study of the response of the rhizosphere microbiome to increased N fertilization. Moreover, its association with soil biochemical properties, such as enzyme activities and chemical compositions, which have been proven indispensable for nutrient transformation and rice yield (Lv et al., 2011; Nannipieri et al., 2018), should receive particular attention.

In this study, we explored the characteristics of the microbial community in the rhizosphere and bulk soil of rice grown in soil that was subjected to three N treatment levels. Increased N fertilizer input altered the bacterial and fungal community structures, which highlighted apparent differences in these structures, in both the rhizosphere and bulk soil. As a result, changes in bacterial community structures followed a generally increasing trend, whereas fungal community structures adhered to a generally decreasing trend, which might be related to the differences in N-acquisition strategies between bacterial and fungal communities (Moreau et al., 2019). Wang et al. (2015) found that oxygen secretion by the rice root system changes the distribution of dissolved oxygen in the rhizosphere, considerably increasing the oxygen concentration in the rhizosphere soil compared with that in the surrounding soil. Considering that aerobic fungi are the primary decomposers of plant biomass (Da Silva et al., 2017), it was therefore not surprising that fungal species diversity was higher and richness was greater in the more oxygenated rhizosphere soil. Conversely, the species diversity and richness of bacteria had decreased significantly with an increase in N fertilization, which is consistent with the findings of Craig et al. (2021). Through the taxonomic annotation of bacterial and fungal communities, we found that N fertilization had a variable impact on the composition of such communities in the soil; notably, increased N fertilization had a greater effect on the composition of fungal communities. By comparing microbial community compositions between the rhizosphere and bulk soil, we found that the rhizosphere effect of rice was mainly reflected in the selective enrichment of fungal communities.

A previous study (Zeng et al., 2016) has shown that N fertilization directly affects species diversity of the microbial community by improving the availability of soil N, whereas alterations to soil pH indirectly affect the microbial composition. In this study, we found that increased N fertilization significantly decreased the pH in both rhizosphere and bulk soil samples. The AN content was significantly lower in soil subjected to N300 treatment than in the N0- and N150-treated soil. Therefore, we confirmed that the excessive application of N fertilizer indeed decreased soil pH in the rice root zone, indirectly affecting the composition of the microbial community. Moreover, the AN content in N300-treated soil was the lowest among the three soil treatment alternatives (Table 2); therefore, we concluded that the excessive application of N fertilizer reduced both the pH and availability of soil N, not only changing the species composition (Figure 3A) but also reducing the species diversity and richness of bacterial communities (Table 1).

Soil nitrification mainly consists of ammonification and nitrite oxidation; the former is the first reaction step in which ammonia is oxidized to nitrite, whereas the latter entails the second step that oxidizes nitrite to nitrate (Daims et al., 2016). Nitrification is mainly accomplished by nitrifying bacteria such as *Nitrosomonas* – a chemoautotrophic bacterium – which oxidizes ammonia to nitrous acid (Purkhold et al., 2003; Marsh et al., 2005), and *Nitrospira*, which is involved in the process of nitrite oxidation (Meincke et al., 1992; Angel et al., 2010). In this study, the relative abundance of *Nitrosomonas* in rhizosphere and bulk soil samples showed a negative correlation with increasing levels of N fertilization, whereas the relative abundance of *Nitrospira* in rhizosphere and bulk soil showed positive and negative correlations with increasing N fertilization, respectively, which may associated with heterotrophic nitrification performed by a wide range of microorganisms, such as heterotrophic bacteria, fungi, and actinomycetes (Zhang Y. et al., 2019; Qiao et al., 2020). These findings, in combination with the results of FAPROTAX analysis, revealed that bacteria with N fixation and nitrification functions – including ammonia and nitrite oxidation – were decreased in bulk soil with increased N application. However, no similar notable change was observed in the rhizosphere, and we infer that this might be related to the rhizosphere effect of rice plants.

N fertilization changes the structure and ecological function of the soil fungal community; FUNGuild is widely used in the study of soil fungal communities and functions (Nguyen et al., 2016; Kolaříková et al., 2017; Leff et al., 2017), classifying fungi according to their ecological functions as pathotrophs, symbiotrophs, or saprotrophs according to their nutritional modes (Nguyen et al., 2016). Previous studies (Bulgarelli et al., 2013; Philippot et al., 2013) have shown that the changes in bacterial community composition from bulk soil to rhizosphere soil are related to bacterial nutritional status, with slow-growing saprophytes occupying most of the space in bulk soil. Conversely, faster-growing bacterial populations based on root-sourced C occupy the majority of the available space in rhizosphere soil. The results of this study could not prove whether the functions of fungi, ranging from bulk to rhizosphere soil, were similar to those of bacteria, but we found that saprophytic fungi were observable in rhizosphere soil, whereas symbiotic fungi were more prevalent in bulk soil. Cavagnaro (2014) found that symbiotic fungi could enhance the ability of crops to obtain nutrients and play an essential role in promoting soil nutrient balance and cycling to improve soil fertility. Compared with that in N0- and N150-treated soil, the relative abundance of symbiotic fungi in the rhizosphere was significantly reduced in soil subjected to N300 treatment. Furthermore, an increasing trend was observed in the abundance of plant pathogens at increased N fertilization levels – N150 and N300 – which was not conducive to plant growth. Although FUNGuild is highly accurate, the ecological functions of many fungi remain unknown. Therefore, further research on and verification of the ecological functions of soil fungi under conditions of increased N fertilization is required.

Jia et al. (2020) found that an increase in soil N content might lead to changes in microbial biomass composition. In this study, the increase in N fertilization had no significant effect on rhizospheric microbial biomass C, N, and P, but a generally decreasing trend was observed. When N fertilizer is added, the amount of inorganic N in soil increases and reacts with soil organic matter, resulting in the formation of heterocyclic N – indole and pyrrole – or phenolic compounds (Thorn and Mikita, 1992). The inorganic N might therefore be unavailable for use in the growth of microorganisms, resulting in insignificant changes in the levels of MBC and MBN. In fact, many studies (Allison et al., 2008; Janssens et al., 2010; Chen et al., 2015) have reported the response of soil microbial biomass to N enrichment, but its influence on soil microorganisms is controversial in terms of both magnitude and effect. The inconsistent results might arise from the heterogeneity of microbes, soil, and plant type variations, differences in N fertilization frequency and methods, and even the duration of N treatment. The addition of N has direct or indirect effects on microbial C and N resources and could therefore promote microorganism growth, whereas soil acidification caused by N enrichment could inhibit the growth of microorganisms. Therefore, the soil microbial biomass response to N fertilization in an intensive agricultural system can be a source of confusion, and the situation is even more complicated for rice grown in flooded soil.

Soil microbiological characteristics are further susceptible to changes in pH, which can affect the activity levels of enzymes participating in soil C and N cycles. However, it can also indirectly affect the secretion of enzymes by affecting the composition of soil microorganisms (Dai et al., 2019). Studies have shown that the addition of N fertilizer enhances the activities of enzymes involved in C and P acquisition but inhibits the activities of N-acquiring enzymes (Jia et al., 2020). In this study, the respective activities of BG and LAP – as C- and N-acquiring enzymes – were the highest in both the rhizosphere and bulk soil samples subjected to N150 treatment, whereas the activity levels of both BG and LAP were significantly reduced, when the N fertilization level was increased to N300. The activity of ACP in the rhizosphere increased significantly at higher N fertilization levels but showed no noticeable change in bulk soil samples, whereas NAG – another N-acquiring enzyme – activity also increased significantly with an increase in N fertilization, which might be related to the decreased soil pH. Sinsabaugh et al. (2008) reported that NAG activity is negatively correlated with soil pH, indicating that increased N fertilizer input results in decreased soil pH, which increases the activity of NAG.

We conducted RDA to further explore the relationship between soil enzyme activity and microbial communities. Our results showed that BG, LAP, and NAG activities were more correlated with the microbial communities in rhizosphere soil. The activities of BG, LAP, NAG, and ACP were all positively correlated with Rhodocyclaceae, indicating that the bacteria might have the function of increasing nutrient-acquisition enzyme activity. Previous studies have shown that Rhodocyclaceae is enriched and considered responsible for denitrification (Chu and Wang, 2013), whereas some strains of Rhodocyclaceae were found to perform nitrate reduction (Ikeda et al., 2013). Ehrenreich et al. (2000) found that Rhodocyclaceae could completely oxidize alkanes. Our results offered no conclusive evidence of the roles of Rhodocyclaceae, but it is most likely related to nitrate reduction and alkane oxidation. Moreover, RDA suggested a positive correlation between fungal communities and soil enzyme activity. Fungi play an essential role in soil nutrient dynamics; not only are they able to compete with decomposing microbial communities for organic N (Phillips et al., 2013; Averill et al., 2014), but fungal species also vary in their capacity to consume different N or P sources, thereby differentially affecting ecosystem-level processes (Tedersoo et al., 2012; Koide et al., 2014). Therefore, changes in the composition of the soil fungal community might lead to changes in enzyme activity, which will have a long-term impact on the N, P, and C cycles in soil. As the availability of inorganic N increases, changes in fungal classification and functional composition are also expected to affect ecosystem processes, including soil C storage, N cycling, and plant productivity (Treseder and Lennon, 2015). Therefore, future research on the function of fungi and the response pattern of fungal communities to N fertilization will be of great significance in improving nutrient utilization and crop productivity in intensive agricultural ecosystems. The fungal species identified in this study – Rhizopodaceae, Nectriaceae, Mortierellaceae, Aspergillaceae, Plectosphaerellaceae, Chaetomiaceae, and Cladosporiaceae – could all be associated with C-, N-, and P-related enzyme activities, providing specific reference value for future research.

## Conclusion

Our study provided evidence that increased N fertilization changes the structure and composition of bacterial and fungal communities in soil. Abundant input of N fertilizer caused soil acidification with adverse effects on the bacterial communities responsible for nitrification and N fixation. Furthermore, plant pathogens were more abundant under conditions of high N fertilization. The properties of soil C, N, and P, as well as soil enzyme activities, were negatively affected by increased N fertilization. We also found that Rhodocyclaceae might be beneficial for improving the activities of BG, NAG, LAP, and ACP. The rhizosphere effect of rice plants was mainly reflected in the selective recruitment of fungal communities, which were more closely related to soil enzyme activities than bacterial communities. This study represents a step toward developing an in-depth understanding of the ways in which shifting bacterial and fungal communities, combined with soil biochemical properties, mediates the effects of and reflects increased N fertilizer input in the rice ecosystem. Our next study will use metagenomic analysis to further evaluate the functional traits of microbial communities, thereby allowing us to better understand the interaction between the microbial communities and improving upon this work.

## Data Availability Statement

The datasets presented in this study can be found in online repositories. The names of the repository/repositories and accession number(s) can be found below: https://www.ncbi.nlm.nih.gov/, PRJNA725973, https://www.ncbi.nlm.nih.gov/, PRJNA725981.

## Author Contributions

DM, HD, and SF conceived the study. HD wrote the manuscript. HD, HS, CC, and AW performed the experiments. HD, HS, and SF performed the statistical analyses. HD, CC, and LJ involved in the field management and soil sampling. All authors discussed the results and commented on the manuscript.

## Conflict of Interest

The authors declare that the research was conducted in the absence of any commercial or financial relationships that could be construed as a potential conflict of interest.

## Publisher’s Note

All claims expressed in this article are solely those of the authors and do not necessarily represent those of their affiliated organizations, or those of the publisher, the editors and the reviewers. Any product that may be evaluated in this article, or claim that may be made by its manufacturer, is not guaranteed or endorsed by the publisher.

## Footnotes

1
https://github.com/PacificBiosciences/barcoding/

2
http://drive5.com/usearch/manual/uchime_algo.html

3
http://www.drive5.com/usearch/

4
www.biocloud.net
